# Efficacy of omega-3 PUFAs in depression: A meta-analysis

**DOI:** 10.1038/s41398-019-0515-5

**Published:** 2019-08-05

**Authors:** Yuhua Liao, Bo Xie, Huimin Zhang, Qian He, Lan Guo, Mehala Subramanieapillai, Beifang Fan, Ciyong Lu, Roger S. McIntyre

**Affiliations:** 1 Department of Psychiatry, Shenzhen Nanshan Center for Chronic Disease Control, Shenzhen, People’s Republic of China; 2 Department of Medical Statistics and Epidemiology, School of Public Health, Sun Yat-sen University, Guangzhou, People’s Republic of China; 3 Mood Disorders Psychopharmacology Unit, University Health Network; Department of Psychiatry, University of Toronto; Institute of Medical Science, University of Toronto; Department of Pharmacology, University of Toronto, Toronto, Ontario Canada

**Keywords:** Clinical pharmacology, Depression

## Abstract

We conducted this meta-analysis of double-blind randomized placebo-controlled trials to estimate the efficacy of omega-3 polyunsaturated fatty acids (PUFAs), especially docosahexaenoic acid (DHA) and eicosapentaenoic acid (EPA), in the improvement of depression. We applied a systematic bibliographic search in PubMed and EMBASE for articles published prior to 20 December 2017. This meta-analysis was performed using RevMan 5.3 and R 3.4.3, and means and standard deviations were calculated in fixed- or random-effects models based on the results of the Q-test. A sensitivity analysis was also conducted to evaluate the stability of the results, and publication bias was evaluated by a funnel plot and Egger’s linear regression analysis. Our search resulted in 180 articles; we analyzed 26 studies, which included 2160 participants. The meta-analysis showed an overall beneficial effect of omega-3 polyunsaturated fatty acids on depression symptoms (SMD = −0.28, *P* = 0.004). Compared with placebo, EPA-pure (=100% EPA) and EPA-major formulations (≥60% EPA) demonstrated clinical benefits with an EPA dosage ≤1 g/d (SMD = −0.50, *P* = 0.003, and SMD = −1.03, *P* = 0.03, respectively), whereas DHA-pure and DHA-major formulations did not exhibit such benefits.

Current evidence supports the finding that omega-3 PUFAs with EPA ≥ 60% at a dosage of ≤1 g/d would have beneficial effects on depression. Further studies are warranted to examine supplementation with omega-3 PUFAs for specific subgroups of subjects with inflammation, severity of depression, and the dose response for both EPA and DHA supplementation.

## Introduction

A growing body of evidence has indicated that omega-3 polyunsaturated fatty acids (omega-3 PUFAs) have been effective in improving depression^1,2^. Supplementation with the two main types of omega-3 PUFAs, eicosapentaenoic acid (EPA)^3^, and docosahexaenoic acid (DHA)^4,5^, has also been found to be effective in reducing symptoms of depression. However, EPA and DHA may play different roles in depression because of their involvement in anti-inflammatory activity and their maintenance of membrane integrity and fluidity, respectively^6^. The different therapeutic effects of EPA and DHA on depression need to be further studied.

The treatment efficacy of supplementation with omega-3 PUFAs in depression is influenced by the proportion and dosage of EPA or DHA. Previous meta-analyses have proposed that PUFAs that are mainly EPA (EPA > 50%^7^, 60%^8^, and 80%^9^ of the dose) have significantly greater efficacy than those that are mainly DHA (DHA > 50%, 60%, and 80% of the dose, respectively), regardless of PUFAs monotherapy or adjuvant use. Some studies have also demonstrated that different dosages of EPA and DHA may result in different levels of efficacy. Recent double-blinded randomized controlled trials (RCTs) indicated that EPA, mostly at dosages of 1 or 2 g/d, was better (than placebo and DHA) as a monotherapy or adjuvant in the treatment of mild to moderate depression and that the ratio of an ‘active’ synergetic effect between EPA and DHA would probably be either 2:1 or 3:1^10–12^. Regarding DHA supplementation, Mischoulon et al. also reported that compared to 4 g/d, greater efficacy was found at 1 g/d and 2 g/d in a single-arm randomized trial on depression^5^.

Given the discrepancy in these methodological aspects, the results would be interpreted differently in each of the previously mentioned studies. However, whether and how EPA and DHA initiate their effects on depression differentially or synergistically with regard to dosage and proportion are still unclear. Therefore, we conducted this meta-analysis to provide an update on the therapeutic effect of omega-3 PUFAs and on the associations between EPA or DHA supplementation and depression, including the effects of the dosage and proportion of EPA or DHA supplementation on depression.

## Materials and methods

### Search strategy

We conducted a systematic bibliographic search for studies that examined the role of omega-3 PUFAs in depression using “major depressive disorder”, “depression”, “fatty acid, omega-3”, and “randomized controlled trial” as key words. We applied our search in the following databases, from inception to 20 December 2017: PubMed and the Ovid version of EMBASE (Supplementary 1). We also searched the references of selected studies and earlier meta-analyses to identify additional potential studies for inclusion in our analysis.

### Study selection

Two independent, trained reviewers screened the identified articles for their relevance by title/abstract and full text. Discrepancies between reviewers were resolved by discussion in all cases and if necessary, by arbitration by a third reviewer. Relevant articles were obtained and included in this review if they had the following characteristics: (1) a double-blind randomized placebo-controlled trial, (2) inclusion of adults with a diagnosis of clinical depression (DSM-III-R/DSM-IV) or depressive symptoms according to validated psychometric instruments (with or without comorbid medical conditions), and (3) clear dosage and duration of EPA and DHA intake. We excluded studies with the following characteristics: (1) depression secondary to other neuropsychiatric disorders and (2) perinatal major depressive disorder.

### Assessment of risk of bias

Two reviewers independently assessed the risk of bias for each study using the criteria outlined in the *Cochrane Handbook for Systematic Reviews of Interventions*^13^. We resolved disagreements by discussion, and we assessed the risk of bias according to the following categories: (1) selection bias, (2) performance bias, (3) detection bias, (4) attrition bias, (5) reporting bias, and (6) other bias.

We judged each potential source of bias as high, low, or unclear risk and have provided a supporting quotation from the study report together with a justification for our judgment in each ‘Risk of bias’ table (Supplementary 2).

### Data extraction

Two reviewers independently assessed and extracted relevant data, including participants’ demographic characteristics, type of clinical diagnosis, psychometric instruments, treatment duration, type and dosage of compound administered, sample size, and psychometric score means and standard deviations (SDs). If the included studies did not report concrete data, we contacted the authors to obtain this information. Our primary analysis used the following hierarchy of psychometric instruments: (1) the Hamilton Rating Scale for Depression (HRSD), (2) the Montgomery-Asberg Depression Rating Scale (MADRS), (3) the Beck Depression Inventory (BDI), and (4) the primary outcome of the selected study.

We divided the omega-3 PUFAs into 4 categories: DHA-pure, DHA-major, EPA-pure, and EPA-major. The DHA- and EPA-pure trials were those in which the omega-3 PUFA supplementation contained only DHA or EPA, respectively. The DHA- and EPA-major categories were those providing higher quantities of DHA (≥60%) or EPA (≥60%), respectively, compared with other omega-3 PUFAs. We further divided the dosages of EPA into ≤1.0 g/d and >1.0 g/d.

### Statistical analysis

We used standardized mean differences as the summary statistic for continuous data by attaining the mean (SD) and sample size (n) of the omega-3 PUFAs and placebo groups. When SDs were not available, we estimated them based on the other statistical parameters reported in the study or requested by authors. All data were analyzed at a single point at the end of the trial. We calculated the Q-statistic to estimate the heterogeneity, and *P* ≤ 0.01 was considered statistically significant for the Q-statistic test. The *I*^2^ statistic was used to quantify heterogeneity, and an *I*^2^-value of 0% indicated no observed heterogeneity, with larger values showing increased heterogeneity. Given the expected heterogeneity, we a priori used a random-effects model. Fixed-effects models would be applied when *I*^*2*^ was <75%. A sensitivity analysis was performed by excluding low-quality studies, trials recruiting participants with particular conditions, or trials with characteristics that were different from those in the other trials. Publication bias was assessed with a funnel plot and Egger’s test. Egger’s linear regression test was used to evaluate asymmetry, and *P* < 0.05 was set as the level of significance. All statistical analyses were performed using RevMan 5.3 (RevMan; The Cochrane Collaboration, Oxford, UK) and R 3.4.3 (R; GitHub, San Francisco, US).

## Results

### Selection of studies

**Fig. 1:**
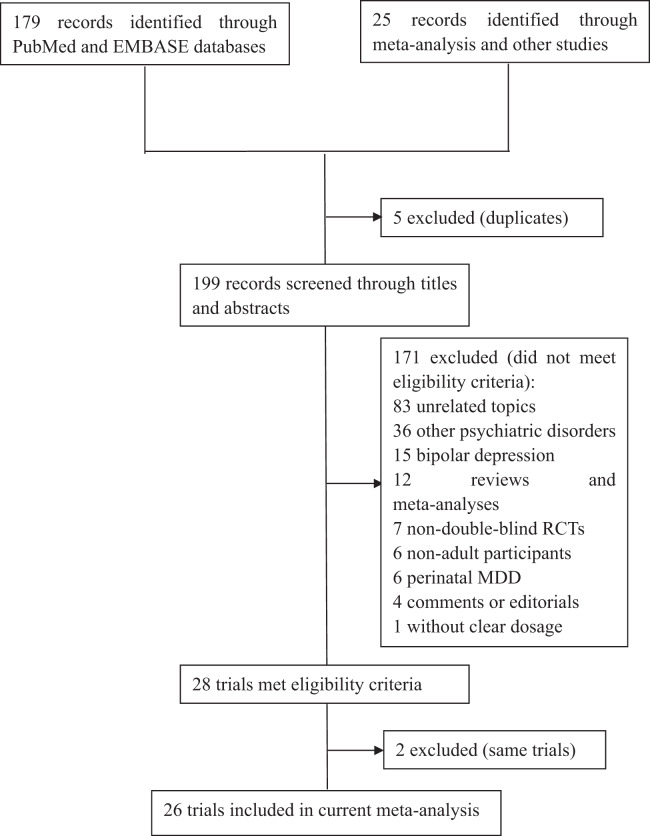
**Flow chart of literature search and study selection**. This figure described the route of studies inclusion. Among 204 researches from database and other studies, there are 26 trials were satisfied for the criteria of our study. MDD major depressive disorder, RCT randomized control trial

An outline of the search strategy is presented in Supplementary 1. From the searches for recently published RCTs in the databases, 179 records were identified. Of these, 173 records were reviewed, and 16 trials met the eligibility criteria. From the searches for systematic reviews or meta-analyses, 25 potential records were identified, and 12 RCTs met the eligibility criteria. Two studies describing results that came from the same trial were removed^14,15^. Ultimately, 26 trials were included in our meta-analysis (Fig. 1), and details of these trials are displayed in Table 1. Among these trials, 12 showed a significant effect of omega-3 PUFAs on the selected rating scales. The risk of bias for all included trials was assessed by the Cochrane methods, and the results can be seen in supplementary 2.

**Table 1:** Systematic review of RCTs of omega-3 PUFAs for depression

Author	Year	Age	Comorbidity	Antidepressants	Clinical diagnosis	Rating scales	Duration	Omega-3 PUFAs	
								EPA	DHA
1. Peet & Horrobin (1 g/d)^16^	2002	18–70	Mixed	With	Without	HDRS-17/ MADRS/BDI	12 weeks	1000 mg/d	/
Peet & Horrobin (2 g/d)	–	–	–	–	–	–	–	2000 mg/d	/
Peet & Horrobin (4 g/d)	–	–	–	–	–	–	–	4000 mg/d	/
2. Nemets et al.^17^	2002	18–75	Mixed	Mixed	DSM-IV	HDRS-24	4 weeks	2000 mg/d	/
3. Su et al.^18^	2003	18–60	Without	Mixed	DSM-IV	HRSD-21	8 weeks	880 mg/d	440 mg/d
4. Marangell et al.^4^	2003	18–65	Without	Without	DSM-IV	HDRS-28/ MADRS	6 weeks	/	2000 mg/d
5. Hallahan et al.^19^	2007	16–64	Without	With	DSM-III	HRSD-17/ BDI	12 weeks	1200 mg/d	900 mg/d
6. Grenyer et al.^20^	2007	18–75	With	Mixed	DSM-IV	HDRS-17	16 weeks	2200 mg/d	600 mg/d
7. Jazayeri et al.^21^	2008	20–59	Without	Without	DSM-IV	HDRS-24	8 weeks	1000 mg/d	/
8. Rogers et al.^22^	2008	18–70	Mixed	Without	Without	BDI/ DASS	12 weeks	630 mg/d	830 mg/d
9. Lucas et al.^23^	2009	44–55	Without	Without	DSM-IV	HAMD-21	8 weeks	1150 mg/d	150 mg/d
10. Carney et al.^24^	2009	adult	With	With	DSM-IV	HAMD-17/ BDI-II	10 weeks	930 mg/d	750 mg/d
11. Mischoulon et al.^11^	2009	18–80	Without	Mixed	DSM-IV	HAMD-17	8 weeks	970 mg/d	/
12. Bot et al.^25^	2010	18–75	With	With	DSM-IV	MADRS	12 weeks	1000 mg/d	/
13. Coryell (1 g/d)	2010	18–55	Mixed	With	DSM-IV	MADRS	6 weeks	740 mg/d	400 mg/d
Coryell (2 g/d)	–	–	–	–	–	–	–	1480 mg/d	800 mg/d
14. Rondannelli et al.^26^	2011	65–95	Mixed	Without	DSM-IV	GDS	8 weeks	1670 mg/d	830 mg/d
15. Tajalizadekhoob et al.^27^	2011	≥65	With	Mixed	Without	GDS-15	6 months	180 mg/d	120 mg/d
16. Antypa et al.^28^	2012	18–65	Without	Mixed	Without	BDI	4 weeks	1740 mg/d	250 mg/d
17. Gertsik et al.^29^	2012	18–65	Mixed	With	DSM-IV	HAMD-21	8 weeks	900 mg/d	200 mg/d
18. Lespérance et al.^30^	2012	≥18	Mixed	With	Without	MADRS	8 weeks	1050 mg/d	150 mg/d
19. Mozaffari-Khosravi et al. (DHA)^3^	2013	18–75	Without	With	DSM-IV	HDRS-17	12 weeks	/	1000 mg/d
Mozaffari-Khosravi (EPA)	–	–	–	–	–	–	–	1000 mg/d	/
20. Gharekhani^31^	2014	Adults	With	Without	Without	BDI-21	12 weeks	1080 mg/d	720 mg/d
21. Mischoulon et al. (DHA)^32^	2015	18–80	Without	Without	DSM-IV	HDRS-17	8 weeks	180 mg/d	900 mg/d
Mischoulon (EPA)	–	–	–	–	–	–	–	1060 mg/d	274 mg/d
22. Park et al.^33^	2015	18–65	Without	With	DSM-IV	HAMD-17	12 weeks	1140 mg/d	600 mg/d
23. Ravi et al.^34^	2016	18–65	With	Without	Without	BDI	8 weeks	720 mg/d	480 mg/d
24. Mazereeuw et al.^35^	2016	45–80	With	Mixed	DSM-IV	HAMD-17/ BDI-II	12 weeks	1200 mg/d	600 mg/d
25. Shinto et al.^36^	2016	18–85	With	With	DSM-IV	MADRS/ BDI	3 months	1950 mg/d	1350 mg/d
26. Rapaport et al. (DHA)^37^	2016	18–80	Without	Mixed	DSM-IV	HAMD-17	8 weeks	180 mg/d	900 mg/d
Rapaport (EPA)	2016	–	–	–	–	–	–	1160 mg/d	260 mg/d

All 26 trials included in this meta-analysis were double-blind, randomized controlled trials. They were summarized as participants’ age, including with or without clinical diagnosis, kinds of rating scales, received therapy dosage and duration

*DSM-IV* Diagnostic and Statistical Manual of Mental Disorders (fourth edition), *DSM-III* Diagnostic and Statistical Manual of Mental Disorders (third edition), *HDRS/HAMD-17/21/24* 17/21/24-item Hamilton Depression Rating Scale, *MADRS* Montgomery Asberg Depression Rating Scale, *BDI* Beck Depression Inventory, *GDS* Geriatric Depression Scale, *omega-3 PUFAs* omega-3 polyunsaturated fatty acids, *EPA* eicosapentaenoic acid, *DHA* docosahexaenoic acid

### Meta-analysis

#### Omega-3 PUFAs efficacy

**Fig. 2:**
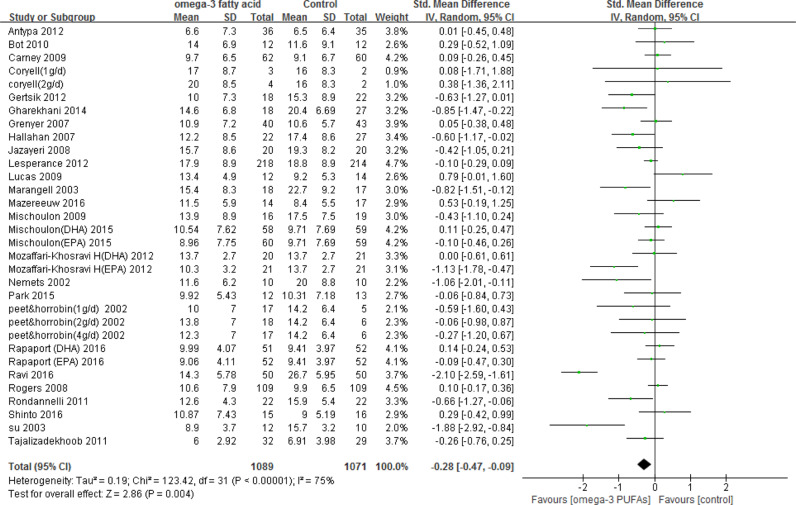
**Forest plot: omega-3 PUFAs vs control**. There was significant effect of omega-3 PUFAs therapy for depression compared to placebo using random effect model. There was also significant evidence of heterogeneity between trials. Size of green plot proportional to weight in meta-analysis. Black lines, show confidence intervals. SD standard deviation, Std. Mean difference standardized mean difference, IV. Random Random (inverse variance heterogeneity), CI confidence interval

A total of 1089 and 1071 subjects were included in the omega-3 PUFAs supplementation group and placebo group, respectively. Analysis was based on the HRSD score in 16 studies^3,4,11,16–21,23,24,29,32,33,35,37^, MADRS score in 4 studies (including one unpublished study, Coryell)^25,30,36^, BDI score in 4 studies^22,28,31,34^ and other scales (The Geriatric Depression Scale, GDS) in 2 studies^26,27^. Overall, omega-3 PUFAs had significant effects on the improvement of depression (Fig. 2); however, the effect sizes were small to modest, and substantial evidence of heterogeneity between studies was detected (*I*² = 75%).

#### DHA and EPA efficacy

Overall, 2 study groups were classified as DHA-pure^3,4^; 3 study groups, DHA-major^22,32,37^; 8 study groups, EPA-pure^3,11,16,17,21,25^; and 16 study groups, EPA-major (including one unpublished study, Coryell)^18,20,23,26–35,37^. For the subgroup analysis of the concentration of omega-3 PUFAs treatments, DHA-pure and DHA-major treatments failed to show significant efficacy in improving depression (SMD = −0.39, *P* = 0.34, and SMD = 0.11, *P* = 0.95, for the fixed-effects and random-effects models, respectively), while EPA-pure and EPA-major treatments were beneficial in improving depression (SMD = −0.48, *P* < 0.001, and SMD = −0.33, *P* = 0.05, for the fixed-effects and random-effects models, respectively).

#### EPA dosage efficacy

The dosage of EPA supplementation ranged from 180 mg/d to 4000 mg/d. We separated the EPA-pure and EPA-major groups into ≤1 g/d and >1 g/d, depending on the EPA dosage. The results indicated that with an EPA dosage ≤1 g/d, the EPA-pure and EPA-major groups demonstrated significant beneficial effects on the improvement of depression (SMD = −0.50, *P* = 0.003, and SMD = −1.03, *P* = 0.03, for the fixed-effects and random-effects models, respectively). We then set a dosage boundary of 1.5 g/d and 2 g/d, but no significant results were detected. Furthermore, we also set a boundary of 50% EPA in omega-3 PUFA supplementation, but we did not observe any of the significant findings for the analyses of different dosage subgroups mentioned above.

#### Publication bias and sensitivity analysis

**Fig. 3:**
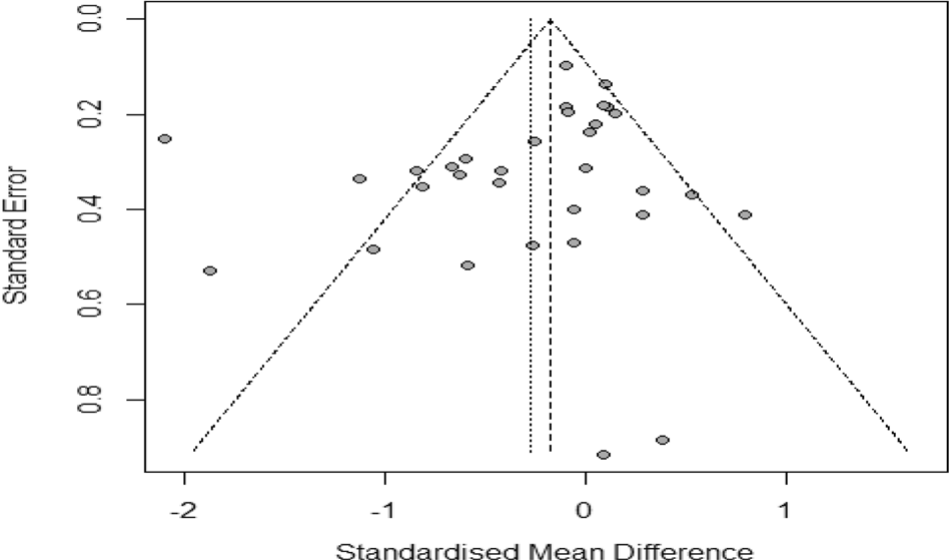
**Funnel plot of effect sizes for clinical trials included in the meta-analysis**. This funnel plot depicts the standardized mean difference of trials versus their standard error. Published trials are depicted as dark circles. The dotted line bracket the 95% CI for the expect results of trials given this estimated underlying effect size

Our funnel plot and statistical test showed no evidence of publication bias (Fig. 3, Egger’s test *P* = 0.17). Running the sensitivity analysis by excluding some high-risk^21,29,31,33^ and unpublished studies (Coryell) had no remarkable effect on the results [SMD = −0.25, 95%CI (−0.46, −0.04), *P* < 0.001, random-effects model]. Excluding studies with specific comorbid chronic physical diseases also did not substantially change the results [SMD = −0.22, 95%CI (−0.38, −0.06), *P* < 0.001, random-effects model].

## Discussion

Herein, we found that omega-3 PUFAs demonstrated a therapeutic effect of −0.28 (95%CI: −0.47, −0.09) on the improvement of depression. Among them, with a dosage of EPA ≤ 1 g/d, the EPA-pure and EPA-major groups demonstrated better effect sizes on the improvement of depression. This effect was in line with those of previous studies^2,9,12^ and may be mediated by the known mechanism of omega-3 PUFAs at the cellular level^38^. In general, omega-3 PUFAs are the essential fatty acids used to produce EPA; from EPA, they then synthesize DHA. However, whether DHA, EPA or both actually contribute to improvements in depression is a topic of considerable debate in current studies. Hence, the subgroup analysis in our meta-analysis demonstrated that only both EPA-pure and EPA-major treatments had favorable effectiveness in depression improvement. This finding was consistent with that of a review by Song et al., which found that the ratio of EPA to DHA that would be most effective for depression was 2:1 or 3:1^12^. Similarly, two meta-analyses considered that the effective proportion of EPA in depression were EPA ≥60%^8^ and ≥80%^9^ respectively. However, the authors examined RCTs including participants with bipolar disorder and Parkinson’s disease and women who were pregnant, which would interfere with the efficacy of omega-3 PUFAs for depression^38^. In contrast, another recent meta-analysis set the lower boundary of the proportion of EPA in omega-3 PUFA supplementation at 50% and showed significant effectiveness^7^. However, that study also included participants with a secondary diagnosis of depression. These results might be related to differences in the metabolism of EPA and DHA. To the best of our knowledge, although EPA is more efficacious with regard to its antidepressant effect, the relationship between EPA and DHA in depression therapy remains unclear. We cannot determine whether the efficacy occurs because of EPA alone or because of an interaction with DHA supplementation. Some possible mechanisms are demonstrated below.

In contrast to DHA, EPA is not highly concentrated in the human brain at steady state. EPA can rapidly enter the brain as a free fatty acid and is not re-acylated into phospholipid membrane stores because it is quickly metabolized and beta-oxidized to act as an effector^39^. Based on this process, in the last decade, the contribution of inflammation to depression has been extensively documented^40,41^, and both DHA and EPA or EPA alone may reduce the occurrence of inflammation (eicosanoids). In the first mechanism, DHA and EPA can lead to decrease in production of proinflammatory cytokines, such as tumor necrosis factor (TNF)-α^42^, interleukin (IL)-1β, IL-2, and IL-6, which are determined by eicosanoid discharge and related to depression. Furthermore, both DHA and EPA can reduce inflammation through their precursor arachidonic acid. DHA and EPA combine with arachidonic acid for amalgamation into membrane-based phospholipids, leading to a decline in both cellular and plasma concentrations of arachidonic acid.

The other possibility is that EPA, but not DHA, can decrease the production of arachidonic acid by inhibiting delta-5-desaturase activity. In the cyclooxygenase enzyme system, EPA may compete with arachidonic acid for phospholipase A_2_ (PLA_2_) and help block the process of proinflammatory eicosanoid synthesis from arachidonic acid (e.g., prostaglandins, thromboxanes, and leukotrienes), prostaglandin E2 and thromboxane B2^38^. In addition, one study determined that individuals with elevated interferon (IFN)-α levels that resulted from chronic hepatitis C always meet operational diagnostic criteria for depression^43^, and a recent RCT pointed out that EPA but not DHA ameliorated IFN-α-induced depression^44^. Overall, although we cannot ignore the contribution of DHA to the depression-related metabolic pathway, the unique antidepressant effects of EPA make it more appealing.

In addition to the anti-inflammatory effect, other mechanisms can explain the advantages of EPA supplementation. First, EPA supplementation has been associated with *N*-acetyl-aspartate increases in the brain, a marker for neuronal homeostasis, suggesting its role as a neuroprotective agent^45^. EPA supplementation also increased the ratio of cerebral phosphomonoesters to phosphodiesters, an indicator of phospholipid turnover, and reversed brain atrophy in a subject with major depressive disorder^46^. Second, EPA is the natural ligand for the peroxisome proliferator-activated receptor gamma (PPARγ) nuclear transcription receptor that downregulates the expression of nuclear factor-kappa B (Nf-kB) and inhibits the neuronal parainflammatory cascades implicated in the pathophysiology of depression^47^. Low concentrations of EPA could bind with very high affinity to all PPARs, whereas DHA binding is too low to be measured, and DHA may provide tonic inhibition of PPARs at high concentrations^48–51^. Third, in the rodent olfactory bulbectomy depression model, EPA treatment normalized depressive behaviors by attenuating prostaglandin E_2_-mediated activation of IL-6, decreasing mRNA expression for corticotrophin-releasing hormone (CRH) and inhibiting hyperactivation of the hypothalamic-pituitary-adrenal (HPA) axis^52^. EPA may also exert a greater neurotrophic effect than DHA, as EPA supplementation has been shown to increase brain-derived neurotrophic factor (BDNF) levels after traumatic brain injury^53,54^. Finally, EPA may be partly related to the brain’s ability to increase dopaminergic and serotonergic neurotransmission^55^.

Based on the significant proportion of EPA supplementation in our results, we also conducted a subgroup analysis to examine the antidepressant effect of its dosage. We found no apparent benefit of a higher dosage (>1 g/d) of either EPA-pure or EPA-major treatment for depression. Depending on the significant difference between EPA dosage groups, our included studies may confirm that the efficacy of EPA is dose dependent. Two assumptions can probably provide explanations. First, in omega-3 PUFAs supplementation for patients with Alzheimer’s disease, EPA could significantly increase to approximately 50 ng/mL in cerebrospinal fluid (CSF) with an intake of 150 mg/d, while DHA increased to only 30 ng/mL based on a dose of 430 mg/d^56^. This finding might suggest a difference in the handling of these two PUFAs during the passage from blood across the blood-brain barrier (BBB) to the central nervous system (CNS) and also serves as a reminder that low doses of dietary EPA intake may be sufficient to have therapeutic effects on depression. Second, a study demonstrated that EPA at high dosages may hamper the activity of CYP2D6 and CYP2A4, which are considered major enzymes in the metabolism of antidepressant drugs (selective serotonin reuptake inhibitors, SSRIs). With the lower boundary of significant EPA supplementation at 720 g/d, we concluded that EPA at proportions of 60% and above in omega-3 PUFAs and a range of 720 mg/d to 1000 mg/d may be more effective in improving depression.

Depended on the efficacy of EPA concentration and dosage, we still cannot neglect the applicable population for this treatment. Considering the patients in augmentation studies may be different from monotherapy through treatment – resistant and severity of depression, we conducted subgroup analysis and only detected the significant effectiveness of augmentation group with EPA-pure (SMD = −0.44, 95% CI −0.81 to −0.06, *P* = 0.02, fixed-effects model, 3 studies and 135 subjects). However, the number of studies and subjects were small, and confidence interval was wide. It may be insufficient to support the efficacy of EPA augmentation. However, there was one meta-analysis demonstrated that EPA was effective in both monotherapy and augmentation^9^, two studies demonstrated a greater antidepressant efficacy of EPA augmentation compared with monotherapy studies^9,21^. Since EPA is highly correlated with inflammation, a proof-of-concept study revealed that EPA supplementation might benefit only major depressive disorder (MDD) subjects with inflammation as part of their syndrome and that it may even be potentially harmful for individuals whose MDD was due to a different physiological disturbance^37^. It may remind us that, in the case of monotherapy and augmentation, the effective supplementation of EPA depends to a large extent on the pathogenesis of the depression patient.

The main limitation of this study was the inability to control for the many potential sources of heterogeneity, including baseline levels of depression, sex, BMI, and baseline plasma omega-3 PUFAs levels. Secondly, we were unable to obtain concrete data describing the subgroup analysis of EPA monotherapy and augmentation sufficiently, as well as the inflammatory biomarkers for all included trials, but the clinical usage of EPA supplementation warrants our attention. In addition, we cannot neglect the efficacy of DHA. In our results, only two RCTs included DHA-pure and DHA-major groups, which was not sufficient to explore the effective proportion and dosage of DHA in omega-3 PUFAs supplementation. Future studies are necessary to address whether DHA or EPA or a combination of the two acting synergistically or antagonistically depends on the specific subgroup of MDD. Finally, the dose effects and the relationship between dietary intake and brain levels of in vivo human omega-3 PUFAs still need to be further studied. The strength of our meta-analysis is that we set strict inclusion criteria for existing RCTs, with a specific focus on excluding certain mental illnesses that would confound the efficacy of omega-3 PUFAs for depression.

Omega-3 PUFAs with formulations containing ≥60% EPA demonstrated antidepressant effects when EPA ≤ 1 g/d. Although many trials support these meta-analysis results, we should nonetheless recognize the heterogeneity among these trials and pay more attention to the proportion and dosage of both DHA and EPA supplementation, including whether they have opposite effects in the case of a 1:1 ratio and how much EPA is effective in improving depression. We note that the long-term efficacy and health effects of omega-3 PUFA supplementation in depression have yet to be elucidated.

## Supplementary information

Supplementary 1 Search strategy

Supplementary 2 Assessment of risk of bias

## References

[1] Appleton, K. M., Sallis, H. M., Perry, R., Ness, A. R. & Churchill R. Omega-3 fatty acids for depression in adults. *Cochrane Database Syst. Rev*. CD004692 (2015). 10.1002/14651858.CD004692.pub4 PMC5321518 26537796

[2] Mocking RJ (2016). Meta-analysis and meta-regression of omega-3 polyunsaturated fatty acid supplementation for major depressive disorder. Transl. Psychiatry.

[3] Mozaffari-Khosravi H, Yassini-Ardakani M, Karamati M, Shariati-Bafghi SE (2013). Eicosapentaenoic acid versus docosahexaenoic acid in mild-to-moderate depression: a randomized, double-blind, placebo-controlled trial. Eur. Neuropsychopharmacol..

[4] Marangell LB (2003). A double-blind, placebo-controlled study of the omega-3 fatty acid docosahexaenoic acid in the treatment of major depression. Am. J. Psychiatry.

[5] Mischoulon D (2008). A double-blind dose-finding pilot study of docosahexaenoic acid (DHA) for major depressive disorder. Eur. Neuropsychopharmacol..

[6] Deacon G, Kettle C, Hayes D, Dennis C, Tucci J (2017). Omega 3 polyunsaturated fatty acids and the treatment of depression. Crit. Rev. Food Sci. Nutr..

[7] Grosso G (2014). Role of omega-3 fatty acids in the treatment of depressive disorders: a comprehensive meta-analysis of randomized clinical trials. PLoS ONE.

[8] Sublette ME, Ellis SP, Geant AL, Mann JJ (2011). Meta-analysis of the effects of eicosapentaenoic acid (EPA) in clinical trials in depression. J. Clin. Psychiatry.

[9] Hallahan B (2016). Efficacy of omega-3 highly unsaturated fatty acids in the treatment of depression. Br. J. Psychiatry.

[10] Peet M, Horrobin DF (2002). A dose-ranging exploratory study of the effects of ethyl-eicosapentaenoate in patients with persistent schizophrenic symptoms. J. Psychiatr. Res..

[11] Mischoulon D (2009). A double-blind, randomized controlled trial of ethyl-eicosapentaenoate for major depressive disorder. J. Clin. Psychiatry.

[12] Song C (2016). The role of omega-3 polyunsaturated fatty acids eicosapentaenoic and docosahexaenoic acids in the treatment of major depression and Alzheimer’s disease: acting separately or synergistically?. Prog. Lipid Res..

[13] Higgins. in *Cochrane Handbook for Systematic Reviews of Interventions (version:5.1.0)* (eds Higgins, J. P. T. & Green, S.) (Chichester, UK: The Cochrane Collaboration & John Wiley & Sons, Ltd. 2011). http://handbook-5-1.cochrane.org/.

[14] Rizzo AM (2012). Comparison between the AA/EPA ratio in depressed and non depressed elderly females: omega-3 fatty acid supplementation correlates with improved symptoms but does not change immunological parameters. Nutr. J..

[15] Rondanelli M (2010). Effect of omega-3 fatty acids supplementation on depressive symptoms and on health-related quality of life in the treatment of elderly women with depression: a double-blind, placebo-controlled, randomized clinical trial. J. Am. Coll. Nutr..

[16] Peet M, Horrobin DF, Group EEMS. (2002). A dose-ranging exploratory study of the effects of ethyl-eicosapentaenoate in patients with persistent schizophrenic symptoms. J. Psychiatr. Res..

[17] Nemets B, Stahl Z, Belmaker RH (2002). Addition of omega-3 fatty acid to maintenance medication treatment for recurrent unipolar depressive disorder. Am. J. Psychiatry.

[18] Su K-P, Huang S-Y, Chiu C-C, Shen WW (2003). Omega-3 fatty acids in major depressive disorder. Eur. Neuropsychopharmacol..

[19] Hallahan B, Hibbeln JR, Davis JM, Garland MR (2007). Omega-3 fatty acid supplementation in patients with recurrent self-harm. Single-centre double-blind randomised controlled trial. Br. J. Psychiatry.

[20] Grenyer BF (2007). Fish oil supplementation in the treatment of major depression: a randomised double-blind placebo-controlled trial. Prog. Neuropsychopharmacol. Biol. Psychiatry.

[21] Jazayeri S (2008). Comparison of therapeutic effects of omega-3 fatty acid eicosapentaenoic acid and fluoxetine, separately and in combination, in major depressive disorder. Aust. N. Z. J. Psychiatry.

[22] Rogers PJ (2008). No effect of n-3 long-chain polyunsaturated fatty acid (EPA and DHA) supplementation on depressed mood and cognitive function: a randomised controlled trial. Br. J. Nutr..

[23] Lucas M, Asselin G, Merette C, Poulin MJ, Dodin S (2009). Ethyl-eicosapentaenoic acid for the treatment of psychological distress and depressive symptoms in middle-aged women: a double-blind, placebo-controlled, randomized clinical trial. Am. J. Clin. Nutr..

[24] Carney RM (2009). Omega-3 augmentation of sertraline in treatment of depression in patients with coronary heart disease: a randomized controlled trial. JAMA.

[25] Bot M (2010). Eicosapentaenoic acid as an add-on to antidepressant medication for co-morbid major depression in patients with diabetes mellitus: a randomized, double-blind placebo-controlled study. J. Affect. Disord..

[26] Rondanelli M (2011). Long chain omega 3 polyunsaturated fatty acids supplementation in the treatment of elderly depression: Effects on depressive symptoms, on phospholipids fatty acids profile and on health-related quality of life. J. Nutr. Health Aging.

[27] Tajalizadekhoob Y (2011). The effect of low-dose omega 3 fatty acids on the treatment of mild to moderate depression in the elderly: a double-blind, randomized, placebo-controlled study. Eur. Arch. Psychiatry Clin. Neurosci..

[28] Antypa N, Smelt AH, Strengholt A, Van, der Does AJ (2012). Effects of omega-3 fatty acid supplementation on mood and emotional information processing in recovered depressed individuals. J. Psychopharmacol..

[29] Gertsik L, Poland RE, Bresee C, Rapaport MH (2012). Omega-3 fatty acid augmentation of citalopram treatment for patients with major depressive disorder. J. Clin. Psychopharmacol..

[30] Lesperance F (2011). The efficacy of omega-3 supplementation for major depression: a randomized controlled trial. J. Clin. Psychiatry.

[31] Dashtikhavidaki S (2014). Effects of omega-3 fatty acids on depression and quality of life in maintenance hemodialysis patients. Am. J. Ther..

[32] Mischoulon D (2015). A double-blind, randomized controlled clinical trial comparing eicosapentaenoic acid versus docosahexaenoic acid for depression. J. Clin. Psychiatry.

[33] Park Y, Park YS, Kim SH, Oh DH, Park YC (2015). Supplementation of n-3 polyunsaturated fatty acids for major depressive disorder: a randomized, double-blind, 12-week, placebo-controlled trial in Korea. Ann. Nutr. Metab..

[34] Ravi S, Khalili H, Abbasian L, Arbabi M, Ghaeli P (2016). Effect of omega-3 fatty acids on depressive symptoms in hiv-positive individuals: a randomized, placebo-controlled clinical trial. Ann. Pharmacother..

[35] Mazereeuw G (2016). Omega-3 fatty acids, depressive symptoms, and cognitive performance in patients with coronary artery disease: analyses from a randomized, double-blind, placebo-controlled trial. J. Clin. Psychopharmacol..

[36] Shinto L (2016). Omega-3 fatty acids for depression in multiple sclerosis: a randomized pilot study. PLoS ONE.

[37] Rapaport MH (2016). Inflammation as a predictive biomarker for response to omega-3 fatty acids in major depressive disorder: a proof-of-concept study. Mol. Psychiatry.

[38] Wani AL, Bhat SA, Ara A (2015). Omega-3 fatty acids and the treatment of depression: a review of scientific evidence. Integr. Med. Res..

[39] Chen CT (2013). The low levels of eicosapentaenoic acid in rat brain phospholipids are maintained via multiple redundant mechanisms[S]. J. Lipid Res..

[40] Członkowska A, Kurkowska-Jastrzębska I (2011). Inflammation and gliosis in neurological diseases-clinical implications. J. Neuroimmunol..

[41] Wee YV (2010). Inflammation in neurological disorders: a help or a hindrance?. Neuroscientist.

[42] Caughey GE, Mantzioris E, Gibson RA, Cleland LG, James MJ (1996). The effect on human tumor necrosis factor alpha and interleukin 1 beta production of diets enriched in n-3 fatty acids from vegetable oil or fish oil. Am. J. Clin. Nutr..

[43] Sarkar S, Schaefer M (2014). Antidepressant pretreatment for the prevention of interferon alfa-associated depression: a systematic review and meta-analysis. Psychosomatics.

[44] Kuan-Pin S (2014). Omega-3 fatty acids in the prevention of interferon-alpha-induced depression: results from a randomized, controlled trial. Biol. Psychiatry.

[45] Frangou S, Lewis M, Wollard J, Simmons A (2007). Preliminary in vivo evidence of increased N-acetyl-aspartate following eicosapentanoic acid treatment in patients with bipolar disorder. J. Psychopharmacol..

[46] Puri BK, Counsell SJ, Hamilton G, Richardson AJ, Horrobin DF (2001). Eicosapentaenoic acid in treatment-resistant depression associated with symptom remission, structural brain changes and reduced neuronal phospholipid turnover. Int. J. Clin. Pract..

[47] Gold PW, Licinio J, Pavlatou MG (2013). Pathological parainflammation and endoplasmic reticulum stress in depression: potential translational targets through the CNS insulin, klotho and PPAR-γ systems. Mol. Psychiatry.

[48] Lin Q, Ruuska SE, Shaw NS, Dong D, Noy N (1999). Ligand selectivity of the peroxisome proliferator-activated receptor alpha. Biochemistry.

[49] Xu HE (1999). Molecular recognition of fatty acids by peroxisome proliferator-activated receptors. Mol. Cell.

[50] Mochizuki K (2006). Selectivity of fatty acid ligands for PPARα which correlates both with binding to cis -element and DNA binding-independent transactivity in Caco-2 cells. Life Sci..

[51] Popeijus HE (2014). Fatty acid chain length and saturation influences PPARα transcriptional activation and repression in HepG2 cells. Mol. Nutr. Food Res..

[52] Song C, Zhang X, Increased M (2009). Phospholipase A2 activity and inflammatory response but decreased nerve growth factor expression in the olfactory bulbectomized rat model of depression: effects of chronic ethyl-eicosapentaenoate treatment. J. Neurosci..

[53] Wu A, Ying Z, Gomezpinilla F (2004). Dietary omega-3 fatty acids normalize BDNF levels, reduce oxidative damage, and counteract learning disability after traumatic brain injury in rats. J. Neurotrauma.

[54] Rao MS, Hattiangady B, Shetty AK (2006). Fetal hippocampal CA3 cell grafts enriched with FGF-2 and BDNF exhibit robust long-term survival and integration and suppress aberrant mossy fiber sprouting in the injured middle-aged hippocampus. Neurobiol. Dis..

[55] Mcnamara RK (2009). Omega-3 fatty acid deficiency during perinatal development increases serotonin turnover in the prefrontal cortex and decreases midbrain tryptophan hydroxylase-2 expression in adult female rats: dissociation from estrogenic effects. J. Psychiatr. Res..

[56] Freund Levi Y (2014). Transfer of omega-3 fatty acids across the blood-brain barrier after dietary supplementation with a docosahexaenoic acid-rich omega-3 fatty acid preparation in patients with Alzheimer’s disease: the OmegAD study. J. Intern. Med..

