# Correction: Efficacy of omega-3 PUFAs in depression: A meta-analysis

**DOI:** 10.1038/s41398-021-01582-6

**Published:** 2021-09-07

**Authors:** Yuhua Liao, Bo Xie, Huimin Zhang, Qian He, Lan Guo, Mehala Subramaniapillai, Beifang Fan, Ciyong Lu, Roger S. McIntyre

**Affiliations:** 1 Department of Psychiatry, Shenzhen Nanshan Center for Chronic Disease Control, Shenzhen, People’s Republic of China; 2 Department of Medical Statistics and Epidemiology, School of Public Health, Sun Yat-sen University, Guangzhou, People’s Republic of China; 3 Mood Disorders Psychopharmacology Unit, University Health Network; Department of Psychiatry, University of Toronto; Institute of Medical Science, University of Toronto; Department of Pharmacology, University of Toronto, Toronto, Ontario Canada

**Keywords:** Clinical pharmacology, Depression

Correction to: *Translational Psychiatry* (2019) 9:190; 10.1038/s41398-019-0515-5; published online 5 August 2019.

The original version of this article unfortunately contained a mistake. After the publication of the article the authors noticed that names of two authors have not been spelled fully: M. Subramaniapillai and R. S. McIntyer, which should appear as Mehala Subramanieapillai and Roger S. McIntyre. The original article has been corrected.

