# Supplementary material for: Efficacy of omega-3 PUFAs in depression: A meta-analysis
Source: Transl Psychiatry. 2019 Aug 5;9:190. doi: 10.1038/s41398-019-0515-5 (PMC6683166; doi:10.1038/s41398-019-0515-5)
Supplement: Supplementary file 1 — Supplementary 1 Search strategy [file 41398_2019_515_MOESM1_ESM.docx]

**Supplementary 1**

**Search strategy**

Search PubMed

("depressive disorder"[MeSH Terms] OR "depression"[MeSH Terms]) OR depression[Title/Abstract] OR depressive disorder[Title/Abstract] OR depressed mood[Title/Abstract]) AND ("fish oils"[MeSH Terms] OR "fatty acids, omega-3"[MeSH Terms] OR Omega-3[Title/Abstract] OR polyunsaturated FA[Title/Abstract] OR fish oil[Title/Abstract] OR EPA[Title/Abstract] OR DHA[Title/Abstract] OR eicosapentaenoic acid[Title/Abstract] OR docosahexaenoic acid[Title/Abstract] OR n-3 fatty acids[Title/Abstract] OR n3 polyunsaturated fatty acids[Title/Abstract]) AND ("Randomized Controlled Trial" [Title/Abstract] OR “clinical trial”[MeSH Terms] OR “randomized placebo-controlled trial” [Title/Abstract])

Search Embase

1. exp depression/

2. depressive disorder.mp.

3. depression.mp.

4. depressed mood.mp.

5. 1 or 2 or 3 or 4

6. fish oil.mp. orexp fish oil/

7. exp omega 3 fatty acid/

8. omega-3.mp.

9. polyunsaturated fatty acid.mp. orexp polyunsaturated fatty acid/

10. expicosapentaenoic acid/ or epa.mp.

11. expdocosahexaenoic acid/ or dha.mp.

12. alpha-linolenic acid.mp. orexplinolenic acid/

13. n-3 fatty acids.mp.

14. n3 polyunsaturated fatty acids.mp.

16. 8 or 9 or 10 or 11 or 12 or 13 or 14

17. randomized controlled trial.mp.

18. 5 and 16 and 17

19. limit 17 to human
