# Supplementary material for: Efficacy of omega-3 PUFAs in depression: A meta-analysis
Source: Transl Psychiatry. 2019 Aug 5;9:190. doi: 10.1038/s41398-019-0515-5 (PMC6683166; doi:10.1038/s41398-019-0515-5)
Supplement: Supplementary file 2 — Supplementary 2 Assessment of risk of bias [file 41398_2019_515_MOESM2_ESM.docx]

**Supplementary 2 Assessment of risk of bias**

| Rogers 2008 | | |
| --- | --- | --- |
| Bias | Author’s judgment | Judgments based |
| Selection bias | Low risk | Randomization was stratified by gender, age and DASS depression score at screen 2, with computer-generated random allocations conducted using (randomly selected) blocks of six or eight. |
| Performance bias | Low risk | The orange oil was included to improve palatability and blinding. If bitten into, their predominant flavour was orange with an oily aftertaste (P.424). Fish and fish oils were not mentioned explicitly in this context. Success of these blinding procedures was evaluated during debriefing by asking participants to identify whether they had received ‘active’ or ‘placebo’ supplements and then asking them what they thought the supplements had contained (P.425). |
| Detection bias | High risk | Participant rated |
| Attrition bias | Low risk | ITT analysis- DASS, BDI,GHQ,STAXI |
| Reporting bias | Low risk | All outcomes reported. |
| Other bias | Low risk | No |
| Peet 2002 | | |
| Bias | Author’s judgment | Judgments based |
| Selection bias | Low risk | “Randomly allocated by PCI clinical services  computer” (P.914) |
| Performance bias | High risk | Participants took the same number of capsules, placebo and intervention capsules were identical in appearance. Participants, researchers and assessors blind to treatment allocation. (P.914) It was unclear if they disguised the fishy taste and no assessment to check concealment. |
| Detection bias | Low risk | HDRS/MADRS - assessors blind to treatment allocation (LOW)  BDI - participant-rated (HIGH) |
| Attrition bias | High risk | HDRS/MADRS/BDI - Not ITT analysis (only 17 participants used in the analysis of placebo group) |
| Reporting bias | Low risk | All outcomes reported. |
| Other bias | Low risk | No |
| Nemets 2002 | | |
| Bias | Author’s judgment | Judgments based |
| Selection bias | Low risk | Participants were randomised according to a random-number table. |
| Performance bias | Low risk | Double-blind. Intervention and placebo capsules were matching, although no attempt to match taste. No participants reported fishy sensations when asked specifically, and debriefing recorded a completely random guess rate by participant and clinician (P.477, 478) |
| Detection bias | Low risk | HDRS - assessors blind to treatment assignment |
| Attrition bias | Low risk | HDRS - 1 participant dropped out, but possible to conduct ITT analysis using LOCF |
| Reporting bias | Low risk | All outcomes reported |
| Other bias | Low risk | No |
| Rondannelli 2011 | | |
| Bias | Author’s judgment | Judgments based |
| Selection bias | Low risk | Bottles for each treatment group were assigned a participant number according to a coded (AB) block randomisation table prepared by an independent statistician. (P.57) |
| Performance bias | Low risk | Investigators were blinded to the randomisation table, the code assignments and the procedure. Bottles of oily preparation were identical for each treatment group and lemon flavour was added to both oils. No participants complained about a fish smell or eructation or made any comment about the contents of the supplement or perception of being in 1 of the 2 groups. (P.58, 60) |
| Detection bias | Low risk | GDS - Investigators and participants blind to treatment |
| Attrition bias | Low risk | GDS - ITT analysis. |
| Reporting bias | Low risk | All outcome reported. |
| Other bias | Low risk | No |
| Tajalizadekhoob 2011 | | |
| Bias | Author’s judgment | Judgments based |
| Selection bias | Low risk | The 66 participants were divided into the placebo and the drug groups using Random Number Generation Method (P.541). |
| Performance bias | Unclear | Two psychologists, not employed by Kahrizak Charity Foundation, assisted in the administration of the GDS-15 and the MMSE assessments (P.541). Hard gelatin capsules had been applied to eliminate the odor and taste of the fish oil in order to keep our study blind. However there was no assessment to check concealment. |
| Detection bias | Low risk | GDS-15/MMSE - Investigators and participants blind to treatment |
| Attrition bias | High risk | Analysis not ITT (P.544) |
| Reporting bias | Low risk | All outcome reported. |
| Other bias | Low risk | No |
| Mozaffai-Khosravi 2012 | | |
| Bias | Author’s judgment | Judgments based |
| Selection bias | Low risk | The allocation sequence was generated by an independent statistician at the Shahid Sadoughi University of Medical Sciences and was given to an independent clinician who dispensed allocated treatments (P.2). |
| Performance bias | Unclear | The randomization sequence was concealed until the last subject completed his final visit (P.2). The blindness has not been assessed. |
| Detection bias | Low risk | All participants were assessed and enrolled at the Bahman neuropsychiatry clinic by trial clinicians who were blinded to treatment allocation. |
| Attrition bias | Low risk | ITT analysis on HDRS. |
| Reporting bias | Low risk | All outcome reported. |
| Other bias | Low risk | No |
| Antypa 2012 | | |
| Bias | Author’s judgment | Judgments based |
| Selection bias | Low risk | Randomization was carried out in blocks of six by an independent person, who had no access to participants’ data (P.739). |
| Performance bias | Low risk | The randomization was conceal for researchers and statisticians. Fish-oil and placebo capsules were lemon-flavoured to maintain the blind. Compliance and success of blinding were checked by a questionnaire after the completion of the study (P.739 and supplementary). |
| Detection bias | High risk | BDI: participant-rated |
| Attrition bias | Low risk | ITT analysis on POMS, LEIDS-R, BDI. |
| Reporting bias | Low risk | All outcome reported. |
| Other bias | Low risk | No |
| Lucas 2009 | | |
| Bias | Author’s judgment | Judgments based |
| Selection bias | Low risk | Researchers responsible for seeing participants allocated next available entry number. Statistician gave randomization list to pharmacy who packaged capsules. (P.642) |
| Performance bias | Low risk | Participants, investigators and staff were blind to treatment assignment until the last participants completed study. (P.642)  Matching placebo with added fish for aftertaste. There was no difference in the number of people guessing their allocation correctly. (P.645) |
| Detection bias | Low risk | HDRS/CGS/HSCL/PGWB- all low. Participants, investigators and staff were blind to treatment assignment until the last participants completed study. |
| Attrition bias | Low risk | ITT analysis - additional information from authors |
| Reporting bias | Low risk | All outcome measures reported. |
| Other bias | Low risk | No |
| Carney 2009 | | |
| Bias | Author’s judgment | Judgments based |
| Selection bias | Low risk | A SAS permuted-block randomization allocation programme. (P.1652) |
| Performance bias | High risk | There was no attempt to mask the fishy taste and no assessment to check concealment. |
| Detection bias | Low risk | Study psychiatrists and nurses were blinded. |
| Attrition bias | Low risk | ITT on BDI and HDRS |
| Reporting bias | Low risk | All major outcomes were reported. |
| Other bias | Low risk | No |
| Bot 2010 | | |
| Bias | Author’s judgment | Judgments based |
| Selection bias | Low risk | Randomization occurred with computer-generated random numbers, performed by an employee of the pharmacy of the VU University Medical Center, who was not involved in the data collection and analysis ([1](#_ENREF_1)). |
| Performance bias | Low risk | Participants and researchers were blinded to treatment allocation until completion of data collection. (P.283) |
| Detection bias | Low risk | MADRS: Research nurse and researchers were blind to treatment allocation until completion of data collection. (P.283) |
| Attrition bias | High risk | MADRS: 1 person in intervention group  lost to follow-up, analysis not ITT (although stated as ITT). (P.284) |
| Reporting bias | Low risk | All depression outcomes reported. (1) |
| Other bias | Low risk | No |
| Su 2003 | | |
| Bias | Author’s judgment | Judgments based |
| Selection bias | Low risk | Random-number sheet generated in Excel. Packages were consecutively numbered according to randomisation schedule by an independent nutritionist. |
| Performance bias | Unclear | Orange flavour was added to the capsules, which were identical to blind the participants (P.268).However there was no assessment to check concealment |
| Detection bias | Unclear | HDRS - unclear whether assessors were blind to treatment allocation |
| Attrition bias | Low risk | HDRS - ITT analysis obtained from author. |
| Reporting bias | Low risk | All outcomes reported. |
| Other bias | Low risk | No |
| Mischoulon 2015 | | |
| Bias | Author’s judgment | Judgments based |
| Selection bias | Low risk | A fixed-block size of 30 participants or a randomly-permuted block size between 6 and 15 participants. P55 |
| Performance bias | Unclear | Flavours added to mask taste but no check to assess blinding. |
| Detection bias | Unclear | Self-reported for mood scales, P55 |
| Attrition bias | High risk | Mood scales - not ITT and > 10% drop out, P55 |
| Reporting bias | High risk | Wellbeing scale and n-3 PUFA blood levels not to be reported |
| Other bias | Low risk | No |
| Grenyer 2007 | | |
| Bias | Author’s judgment | Judgments based |
| Selection bias | Low risk | Randomisation and capsule packing performed externally. (P.1394) |
| Performance bias | High risk | Participants, clinicians and researchers were blind to allocation. Identical placebo and capsules odorless, however when checked the majority (90% fish oil group, 64% placebo group) of participants correctly guessed their group. (P.1395) |
| Detection bias | Low risk | HDRS - clinician-rated: physicians were blinded to allocation. |
| Attrition bias | High risk | ITT analysis conducted but 28% dropped out. |
| Reporting bias | Low risk | All relevant outcomes reported. |
| Other bias | Low risk | No |
| Gertsik 2012 | | |
| Bias | Author’s judgment | Judgments based |
| Selection bias | Unclear | “Block randomised by sex to receive citalopram. Half of the subjects also received omega-3 and the other half received placebo” but method of sequence generation not reported. (P.62) |
| Performance bias | High risk | The study was described as “masked” but not clear who was blinded (P.61). It was unclear if the fishy taste was disguised and no assessment to check concealment. |
| Detection bias | Unclear | HDRS, MADRS and BDI - all unclear -  The study was described as “masked” but it was unclear who was blinded. (P.61) |
| Attrition bias | High risk | HDRS, MADRS, BDI - Analysis not ITT and > 10% missing data. |
| Reporting bias | High risk | No protocol to check for additional outcome measures. CGI, PGI not reported. |
| Other bias | Low risk | No |
| Mischoulon 2009 | | |
| Bias | Author’s judgment | Judgments based |
| Selection bias | Low risk | Randomisation performed by research pharmacy using [www.randomization.com](http://www.randomization.com) (P.1637) |
| Performance bias | High risk | It was unclear if the fishy taste was disguised and no assessment to check concealment. |
| Detection bias | Low risk | HDRS - Study clinicians remained blind to assignment for duration of study |
| Attrition bias | Low risk | HDRS - ITT numbers obtained through correspondence with author. |
| Reporting bias | High risk | All primary outcome measures reported. QLESQ was a planned outcome and measured but not analysed or reported. |
| Other bias | Low risk | No |
| Marangell 2003 | | |
| Bias | Author’s judgment | Judgments based |
| Selection bias | Unclear | Not reported. |
| Performance bias | Unclear | Not reported. |
| Detection bias | Unclear | MADRS/HDRS – unclear whether assessor was blinded to treatment. |
| Attrition bias | High risk | MADRS/HDRS - not ITT analysis |
| Reporting bias | Unclear | No protocol available to check prespecified outcome measures |
| Other bias | Low risk | No |
| Park 2015 | | |
| Bias | Author’s judgment | Judgments based |
| Selection bias | Low risk | Independent statistician, computer-generated randomisation scheme allowing for randomisation blocks, P143 |
| Performance bias | High risk | No attempt to mask flavour or check blinding, P142 |
| Detection bias | Low risk | HDRS scores were measured by psychiatrist who was blinded to treatment groups |
| Attrition bias | High risk | HDRS - > 10% missing in the overall sample and not ITT analysis |
| Reporting bias | Low risk | All outcomes reported. |
| Other bias | High risk | Significant baseline imbalance for mood disorders, P144 |
| Coryell 2010 | | |
| Bias | Author’s judgment | Judgments based |
| Selection bias | Unclear | Researcher was blind to allocation, research nurse was not blind to allocation. |
| Performance bias | Unclear | Researcher was blinded, research nurse was not blinded, and both had contact with participants. Possible attempts to check blinding, but no data available. |
| Detection bias | Low risk | Outcome assessments made by researcher, and researcher was blinded. |
| Attrition bias | Low risk | Complete MADRS outcome data, and ITT analysis. |
| Reporting bias | High risk | Data not published. |
| Other bias | Low risk | No |
| Gharekhani 2014 | | |
| Bias | Author’s judgment | Judgments based |
| Selection bias | Unclear | No reported allocation concealment. |
| Performance bias | High risk | No masking of fishy taste and no assessment to check concealment. |
| Detection bias | High risk | BDI (self-report). |
| Attrition bias | High risk | BDI - ITT data provided by authors, but >  10% dropout. |
| Reporting bias | Low risk | All outcomes reported. |
| Other bias | Low risk | No |
| Jazayeri 2008 | | |
| Bias | Author’s judgment | Judgments based |
| Selection bias | Unclear | “Prearranged block randomization” (P.193)  but unclear how sequence was generated  Permuted-block randomization. |
| Performance bias | High risk | “Double dummy” placebo technique used to blind participants. However, no steps taken to mask fish taste and no assessment to check concealment (P.194 - 5). |
| Detection bias | Low risk | HDRS - physicians blind to treatment allocation. |
| Attrition bias | High risk | HDRS - ITT data provided by authors, > 10% dropout from each group. |
| Reporting bias | High risk | Some outcomes not yet published. |
| Other bias | Low risk | No |
| Lespérance 2012 | | |
| Bias | Author’s judgment | Judgments based |
| Selection bias | Low risk | Computer-generated, randomly permuted blocks of 2 and 4, stratified by site and baseline antidepressant use/non-use. (P.1056) |
| Performance bias | Low risk | Study research personnel and participants were blinded. 2% fish oil was added to placebo to control for fishy aftertaste. James’ blinding index used to check blinding of treatment allocation. (P. 1056) |
| Detection bias | Low risk | IDS-SR and MADRS both low- study psychiatrists, personnel were blinded. |
| Attrition bias | High risk | ITT analysis and although similar dropouts in each group, > 10% dropout. |
| Reporting bias | Low risk | All outcomes reported. |
| Other bias | Low risk | No |
| Hallahan 2007 | | |
| Bias | Author’s judgment | Judgments based |
| Selection bias | Low risk | An independent colleague dispensed either active or placebo capsules according to a computer-generated list. |
| Performance bias | Unclear | The code was only revealed to the researchers once data collection was complete. Placebo contained 99% corn oil and a 1% EPA/DHA mixture. This ensured a degree of equality in the incidence of ‘fishy breath’. |
| Detection bias | Low risk | HDRS- psychiatrists and participants were blinded |
| Attrition bias | High risk | Not ITT analysis |
| Reporting bias | Low risk | All outcomes reported. |
| Other bias | Low risk | No |
| Saeedeh 2008 | | |
| Bias | Author’s judgment | Judgments based |
| Selection bias | Low risk | Microsoft Office Excel software was used to generate numbers and randomize the patients to the groups (P.798). |
| Performance bias | Unclear risk | All patients as well as the interviewer and physicians were blinded regarding the intervention types during the study period (P.798). Blindness assessments had not been mentioned. |
| Detection bias | High risk | BDI-II: participant-rated |
| Attrition bias | High risk | ITT analysis had not been applied. |
| Reporting bias | Low risk | All outcomes reported. |
| Other bias | Low risk | No |
| Mazereeuw 2016 | | |
| Bias | Author’s judgment | Judgments based |
| Selection bias | Low risk | A block randomization code was independently computer generated at Sunnybrook Health Science Centre pharmacy (P.437). |
| Performance bias | Low risk | All study personnel remained blind to treatment allocation until the database was “locked”. The n-3 PUFA and placebo capsules were similar in appearance (dark brown) and taste (lemon-lime flavoring). However, no assessments were used to evaluate blindness (P.437). |
| Detection bias | Low risk | Blindness assessment on HAMD and BDI-II |
| Attrition bias | Low risk | ITT analysis on HAMD and BDI-II |
| Reporting bias | Low risk | All outcomes reported. |
| Other bias | Low risk | No |
| Shinto 2016 | | |
| Bias | Author’s judgment | Judgments based |
| Selection bias | Low risk | Participants were randomized to receive either placebo or omega-3 fatty acids by an independent pharmacist who dispensed either active or placebo capsules according to a computer generated randomization list (P.3). |
| Performance bias | Low risk | Participants were randomized to receive either placebo or omega-3 fatty acids by an independent pharmacist. Participants randomized to the placebo group received capsules that contained soybean oil with 1% fish oil so that it was flavored to taste and smell similar to the fish oil capsules (P.3). When asked about treatment assignment at the end of the study the majority reported no knowledge of treatment assignment, research staff (100%), placebo subjects (75%), and omega-3 FA subjects (80%). |
| Detection bias | Low risk | The Department of Psychiatry at OHSU supervised the training and administration of the SCID and MADRS. |
| Attrition bias | High risk | Compliance by pill count was > 90% for both groups. But ITT analysis has not been applied. |
| Reporting bias | Low risk | All outcomes reported. |
| Other bias | Low risk | No |
| Rapaport 2016 | | |
| Bias | Author’s judgment | Judgments based |
| Selection bias | Unclear | Subjects were randomized in a double-blind 1:1:1 manner to three groups. Randomization method was unknown. |
| Performance bias | Unclear | Unclear attempt to mask flavour or check blinding. |
| Detection bias | Unclear | Unclear attempt to assessment blindness. |
| Attrition bias | Low risk | ITT analysis on HAMD-17 |
| Reporting bias | Low risk | All outcomes reported. |
| Other bias | Low risk | No |

Abbreviation: BDI: Beck depression inventory; CGI: Clinical Global Impression Scale; CGS: Clinical Global Impression Severity Scale; DHA: docosahexaenoic acid; DASS: Depression Anxiety Stress Scale; EPA: eicosapentaenoic acid; GDS: geriatric depression scale; GHQ: General Health Questionnaire; HSCL: Hopkins symptom checklist depression scale; IDS-SR: Patient’s Global Improvement Scale; ITT: intention-to-treat; HDRS/ HAMD: Hamilton depression rating scale; LOCF: last observation carried forward; LEIDS-R: Leiden Index of Depression Sensitivity-Revised; MADRS: Montgomery-Asberg depression rating scale; MMSE: mini mental state examination; PGI: Patient’s Global Improvement Scale; PGWB: psychological general well being; POMS: Profile of Mood States; QLESQ: quality of life enjoyment and satisfaction questionnaire; SAS: Statistics Analysis System; SCID: structured clinical interview (depression); STAXI: State-Traint Anger Expression Inventory

Reference

1. Mocking RJ, Assies J, Bot M, Jansen EH, Schene AH, Pouwer F. Biological effects of add-on eicosapentaenoic acid supplementation in diabetes mellitus and co-morbid depression: a randomized controlled trial. PLoS One. 2012;7(11):e49431.
